# Molecular Characterization of α- and β-Thalassaemia among Malay Patients

**DOI:** 10.3390/ijms15058835

**Published:** 2014-05-19

**Authors:** Nur Fatihah Mohd Yatim, Masitah Abd. Rahim, Kavitha Menon, Faisal Muti Al-Hassan, Rahimah Ahmad, Anita Bhajan Manocha, Mohamed Saleem, Badrul Hisham Yahaya

**Affiliations:** 1 Regenerative Medicine Cluster, Advanced Medical and Dental Institute, Universiti Sains Malaysia, Bertam, Kepala Batas, Penang 13200, Malaysia; E-Mails: teaha_101@yahoo.com.my (N.F.M.Y.); masitahrahim@yahoo.com (M.A.R.); mohamedsaleem@amdi.usm.edu.my (M.S.); 2 Healthy Lifestyle Cluster, Advanced Medical and Dental Institute (AMDI), Universiti Sains Malaysia, Kepala Batas, Penang 13200, Malaysia; E-Mail: kavitha@amdi.usm.edu.my; 3 Allianze University College of Medical Sciences (AUCMS), Waziria Medical Square, Jalan Bertam 2, Mukim 6, Kepala Batas, Penang 13200, Malaysia; E-Mail: alhassanfaisal@ymail.com; 4 Haematology Unit, Cancer Research Centre, Institute for Medical Research, Jalan Pahang, Kuala Lumpur 50588, Malaysia; E-Mail: rahimah@imr.gov.my; 5 Department of Medicine, Hospital Seberang Jaya Jalan Tun Hussein Onn, Seberang Prai 13700, Malaysia; E-Mail: amanocha@ppg.moh.gov.my

**Keywords:** α-thalassaemia, β-thalassaemia, Malay, Penang

## Abstract

Both α- and β-thalassaemia syndromes are public health problems in the multi-ethnic population of Malaysia. To molecularly characterise the α- and β-thalassaemia deletions and mutations among Malays from Penang, Gap-PCR and multiplexed amplification refractory mutation systems were used to study 13 α-thalassaemia determinants and 20 β-thalassaemia mutations in 28 and 40 unrelated Malays, respectively. Four α-thalassaemia deletions and mutations were demonstrated. −−^SEA^ deletion and α^CS^α accounted for more than 70% of the α-thalassaemia alleles. Out of the 20 β-thalassaemia alleles studied, nine different β-thalassaemia mutations were identified of which β^E^ accounted for more than 40%. We concluded that the highest prevalence of (α- and β-thalassaemia alleles in the Malays from Penang are −−^SEA^ deletion and β^E^ mutation, respectively.

## 1. Introduction

Thalassaemias are autosomal recessive disorders characterised by quantitative defects in globin chain synthesis. These biosynthetic defects can be classified according to the globin chain or chains involved in deficient synthesis. Best characterised are α- and β-thalassaemia [1], which result from defective synthesis of alpha and beta chains, respectively [2].

Thalassaemia is one of the most common inherited disorders of haemoglobin [3] and is currently a public health problem affecting Malaysia’s multi-ethnic population [4]. Studies have shown that 3%–5% of the 28.3 million people in Malaysia are carriers of the thalassaemia gene [5,6]. The major ethnic groups are Bumiputras who make up the majority, followed by the Chinese and Indians [7]. In 2010, there were 4768 registered thalassaemia patients in Malaysia and between 600,000 and 1,000,000 carriers of the thalassaemia trait [4]. Current statistics show that 1 in 20 Malaysians are carriers of the β-thalassaemia trait [8]. It was further estimated that 120–350 infants are born each year with transfusion-dependent thalassaemia [8].

In most of the existing clinical set ups in Penang, the diagnosis of thalassaemia is based on data obtained from clinical findings, blood picture and haemoglobin analysis rather than molecular characterisation. It would be prudent to perform molecular characterisation for the genotype before clinical management of thalassaemia is begun. Characterising the most common α- and β-thalassaemia mutations in the Malay population should make the subsequent diagnostic approach simpler, more cost effective and quicker by focusing on the small number of ethnically predominant alleles instead of a wide range of rare alleles. Since the frequencies of α- and β-thalassaemia alleles vary considerably with geographic location and ethnic group, this study was performed to characterise α- and β-thalassaemia mutations at the molecular level among Malay patients from Penang, a northern state of Peninsula Malaysia.

## 2. Results

**Figure 1.**
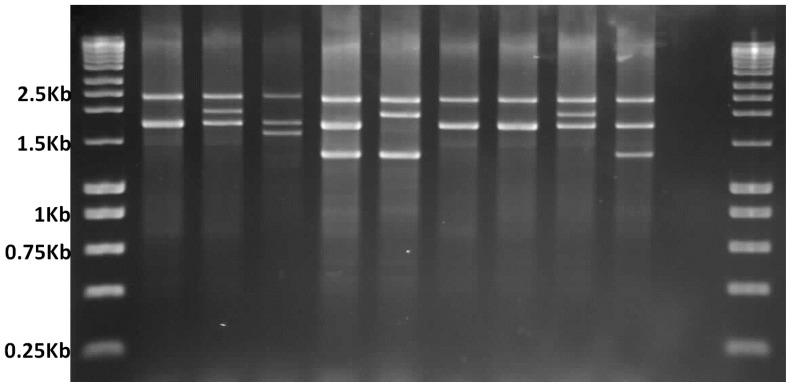
Multiplex-Gap PCR genotype analysis of the α-globin gene cluster on agarose gel electrophoresis. Lanes 1 and 12 (left to right) show DNA marker ladders; 2350 bp band on lane 2 depicts the internal control and 1800 bp band indicates the presence of normal α2 globin gene. Lanes 3–5 are positive controls for heterozygous −α^3.7^ deletion, −α^4.2^ deletion, and −−^SEA^ deletion; Lanes 6–10 are patients where lane 6 shows compound heterozygosity for −α^3.7^ and −−^SEA^ deletions; Lanes 7 and 8 “none of the deletion tested present”; Lane 9 −α^3.7^ deletion; Lane 10 −−^SEA^ deletion; and Lane 11 is non-template control.

**Figure 2.**
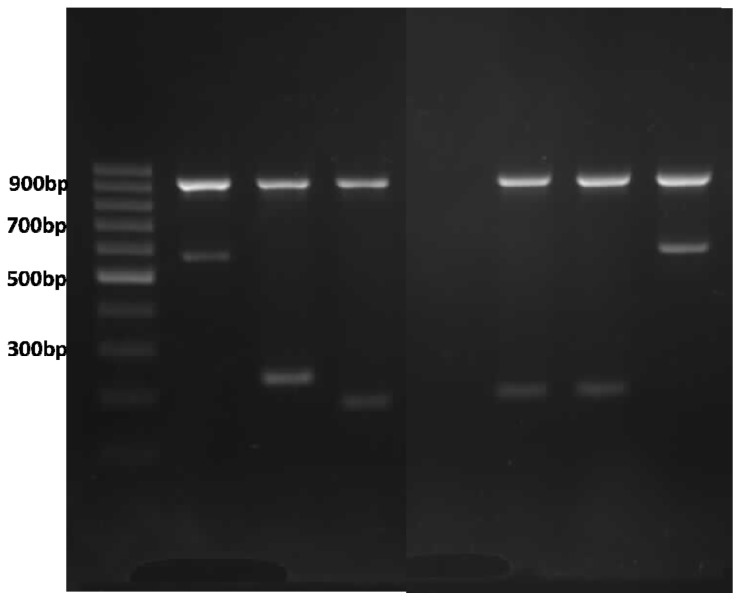
Multiplex ARMS PCR genotype analysis of α globin gene cluster on agarose gel electrophoresis. Lane 1 ladder; Lanes 2–4 are positive controls for Cd 59 (G > A), Cd 125 (T > C), and term Cd TAA > CAA (Hb CS) with their respective bands; Lane 5 non-template control; Lanes 6 and 7 Hb CS; and Lane 8 Cd 59 (G > A); 930 bp bands on Lanes 2–4 and 6–8 are internal control bands amplifying a segment of 3′ UTR of *LIS1* gene.

A total of 56 α-globin gene haplotypes from Malay patients from Penang were characterised. As summarized in Table 1, two forms of deletions and two forms of mutations were detected out of the 13 different α-thalassaemia determinants tested. The most frequent amongst them was −−^SEA^, demonstrated on 20 α-globin haplotypes with a prevalence of 35.7%. This was followed by the chain termination mutant (α^CS^α) that was found on 30.3% (17/56) of α-globin gene haplotypes studied. The remaining two alleles, −α^3.7^ and Cd 59, were less frequently observed. These four determinants, along with the normal haplotype (αα), were observed to interact in six different genotypes as depicted in Table 2. *A posteriori* high frequency of −−^SEA^/α^CS^α made up 46% of the genotypes. About 21% (6/28) were compound heterozygous for the −−^SEA^/−α^3.7^α. Surprisingly, 4/28 (14.3%) of patients were compound heterozygous for the α^Cd 59^α/ α^CS^α genotype; and 2/28 (7.1%) of patients were carriers of the α^Cd 59^α/αα genotype. It was observed that 2/28 (7.1%) of patients were homozygous for the −α^3.7^/−α^3.7^ genotype, while 1/28 (3.6%) of patients were heterozygous for the −−^SEA^/αα genotype. The other deletion and non-deletion α-thalassaemia gene defects screened were not detected using the above test methods. Some of the representative agarose gel electrophoresis are shown in Figures 1 and 2 for multiplexed ARMS-PCR and Gap-PCR, respectively.

**Figure 3.**
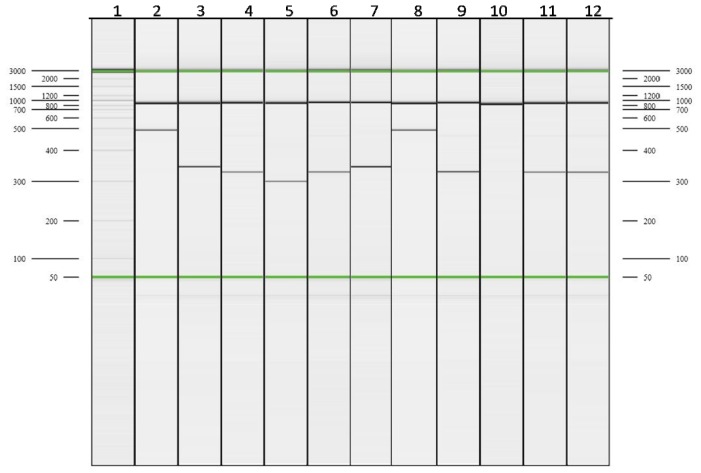
Capillary electrophoresis (CE) electropherogram for multiplex ARMS-A of β globin gene cluster. Lanes 2–5 are the positive controls for 41/41 (−TTCT), IVS 1-5 (G > C), Cd 26 (G > A), and Cd 17 (A > T) respectively. Lanes 6, 9, 11, and 12 show Cd 26 (G > A); Lane 10 shows “none of the mutations tested present”; and Lanes 7 and 8 show the presence of IVS 1-5 (G > C).

A total of 40 Malay patients with β-thalassaemia syndromes were enrolled in this study. From the 80 β globin genes studied, nine different mutations were detected on 65 chromosomes. As depicted in Table 3, nine patients were heterozygous for β-thalassaemia, 28 patients were compound heterozygous for β-thalassaemia and three patients remained uncharacterised by this procedure using the 20 different mutations described above. As summarised in Table 4, Cd 26 (G > A) and IVS 1-5 (G > C) were the two most common mutations observed and they accounted for a little over 60% of the patients. IVS 1-1 (G > T) and Cd 41/42 (−TTCT) had equal prevalence cumulatively accounting for a quarter of all the mutations observed. The remaining alleles, Cd 15 (G > A), Cd 17 (A > T), Cd 19 (A > G), Cd 8/9 (+G) and −28 (A > G) were rare alleles and were observed either on two chromosomes or on singletons. Capillary gel electrophoresis for the common β globin gene alleles is shown in Figure 3.

## 3. Discussion

The reported frequencies of α- and β-thalassaemia mutations and deletions vary considerably between ethnic groups and with geographic locations [7,8]. Consequently, this study was performed to characterise the α- and β-thalassaemia determinants in the mainland of Penang where the majority of the population is Malay. Samples were taken from Malay patients who were previously diagnosed with α- or β-thalassaemia by the HPLC method at the Hospital Seberang Jaya (HSJ), Penang. All the 68 patients had a mean corpuscular volume below 80 fL and mean corpuscular haemoglobin less than 27 pg.

The present study showed −−^SEA^ to be the most prevalent α-thalassaemia determinant in Penang, with a prevalence of 35%, followed by α^CS^α (30%) and −α^3.7^ (18%). The inflated prevalence observed here clearly contradicts the nationwide prevalence reported for α-thalassaemia amongst Malays at large [9–11]. Several studies have shown plausible evidence that −−^SEA^ *cis* gene deletion as the most common amongst Malaysian Chinese, while this deletion is not as common as the −α^3.7^ allele in Malays [3,9–11]. On average, throughout the population, the −−^SEA^ deletion is characteristically four times more common in Malaysian Chinese than in Malays [3,10,11]. Moreover, Hb CS which was observed at a prevalence of 30% in this study, by contrast, has a nationwide frequency ranging between 1.1% and 3.6% (95%CI) in the ethnic Malays [11]. The most likely explanation for this remarkable overrepresentation of the α-thalassaemia genes in this study is the non-random nature of samples, representing symptomatic patients attending for regular follow-ups at Hospital Seberang Jaya. As is confirmed here, nearly 82% of these patients had HbH disease—out of which over 46% of patients had non-deletional HbH disease (−−^SEA^/α^CS^α); and 21% of patients had deletional HbH disease (−α^3.7^/−−^SEA^). Another 14% of subjects had genotype α^Cd 59^α/α^CS^α presenting phenotypically as thalassaemia intermedia or HbH disease, presumably because haplotype α^Cd 59^α has α^0^ phenotype [2]. Therefore, the contact clinical sampling method adopted and the small sample size used for characterising the α-thalassaemia gene are likely to be the two main reasons for the discordance in allelic prevalence reported here. Furthermore, since no clinical disturbances are generally associated with either α^+^-thalassaemia homozygotes (−α/−α) or α^0^ heterozygotes (−−/αα) these benign α-thalassaemia minor individuals are not well represented and neither are the alleles carried by them. The aforementioned genotypes are incidentally diagnosed during routine haematological examinations or as a result of family screening to diagnose a symptomatic thalassaemia in a relative. It is therefore necessary to obtain a simple random sample that is representative of the Malay subpopulation in the Penang district to consolidate these observations. The results were similar to a study from the Guangdong Province in Southern China. It was noted that the −−^SEA^ deletion was the most common mutation detected (48.54%) followed by the −α^3.7^ deletions [12].

A diverse spectrum of nine β-thalassaemia alleles (Table 4) were demonstrated. Cd 26 (CAG > AAG), IVS I-5 (G > C), IVS I-1 (G > T) and Cd 41/42 (−TTCT) accounted for 86% of the mutations characterised. The highly skewed five rare alleles accounted for the remaining 14%. The β^E(26Glu→Lys)^ mutation is consistently the most common mutation among Malays, while Cd 41/42 (−TTCT) is more specific and widespread in the Malaysian Chinese [9,13]. Invariably, Hb E (β^26Glu→Lys^) observed in this analysis at a prevalence of 35% is the most common β globin gene defect among Malays from Penang. This high prevalence is consistent throughout the Malay sub-population, though considerable variation exists from one geographic location to another [13–16]. Characterisation of β-thalassaemia alleles on a contemporary multiethnic sample by Hassan *et al.* and George *et al.* revealed that HbE among the Malay ethnic group were 23% and 28.8%, respectively [9,13]. A similar study conducted a decade earlier by Tan *et al.* demonstrated the frequency of HbE in ethnic Malay was 19% [6].

It is observed here that the prevalence of IVS 1-5 (G > C) among Malays from Penang is three-fold lower than the most common HbE allele. By contrast, Hassan *et al.* reported an equal distribution of both alleles among Malays throughout Malaysia [13], while Tan *et al.* reported *a posteriori* elevation of the former by an approximate ratio of 2:1 [6]. This empirical increase in the prevalence of HbE among Malays from Penang could be attributed to its clinical severity as the mutation is known to be associated with the β^0^-thalassaemia phenotype, especially when the gene is in compound heterozygosity with a second β-thalassaemia allele (β^E^/β^0^ or β^E^/β^+^). In fact, all the 23 compound heterozygous patients with β^E^ (Table 4) characterised here had a history of red cell transfusions at variable frequencies. This exemplified the clinical severity and disease heterogeneity of the disease when the Hb E allele is in *transposition* with another β mutation. Pathologically, this point mutation, commonly known as haemoglobin variant, simultaneously produces a structural and a quantitative defect in globin chain synthesis; hence, it is better known as thalassaemia haemoglobinopathy [17].

The third most common β-thalassaemia mutation characterised here is IVS I-1 (G > T). As with the other observations, this mutation is prominently only found in Malays, among whom it has reached a high heterozygous carrier frequency and has given rise to a β^0^ phenotype [12,14–16].

A relatively high prevalence of Cd 41/42 (−TTCT) is observed in Penang. This allele is prominent in the Malaysian-Chinese and less frequent amongst ethnic Malays in the general population [13,14]. This remarkable incongruity could be due to miscegenation between Malays and Chinese in Penang undergoing a demographic process that changes the allelic boundaries. This phenomenon could be acting on the distribution of many other thalassaemia alleles in this multiethnic subpopulation as Malaysians live side-by-side, breaking the norms of endogamy. Even a low level of gene flow over generations can lead to substantial changes, producing a heterogeneous pattern of allele frequencies.

Interestingly, Cd 15 (G-A) and Cd 8/9, which are usually the rare types of β-thalassaemia mutations among Malays, are also observed in this study. In addition, Cd 15 (G-A) was found in the Thai population, at a prevalence of 0.3% [18]. In Malaysia, Cd 8/9 has been found in the Kedayan population, while Cd 15 (G-A) was found in Malaysian Indian patients but at a very low frequency [13].

## 4. Experimental Section

### 4.1. Sample Collections

A total of 68 Malay patients attending Hospital Seberang Jaya (HSJ), a part of the northern state of Peninsular Malaysia, were enrolled. A subset of 28 patients positive for HbH on high performance liquid chromatography (HPLC) or positive for HbH inclusion bodies on 1% Brilliant Cresyl blue (BCB) were recruited for α-thalassaemia gene characterisation. A second subset of 40 patients with hypochromic microcytic anaemia and elevated HbA_2_ above 3.5% were enrolled for β-thalassaemia mutation screening. Prior ethical approval was obtained from the Human Research Ethics Committee (JEPeM), Universiti Sains Malaysia and Research and Ethics Committee (MREC, Kuala Lumpur, Malaysia), Ministry of Health. Approximately 2 mL of whole blood was collected into EDTA vacutainer (Bio Lab, Shah Alam, Malaysia) from each patient after obtaining informed consent.

### 4.2. DNA Extraction

Genomic DNA was extracted from 200 μL whole blood using a commercially available DNA extraction kit, QIAamp DNA Blood Mini Kit (Qiagen GmbH, Hilden, Germany). The concentration of DNA was determined by NanoDrop Spectrophotometer ND-1000 (NanoDrop Technologies, Wilmington, DE, USA). The extraction was carried out according to the manufacturer’s instructions. To perform PCR in this study, a concentration of DNA around 50 ng/μL was required.

### 4.3. Molecular Analysis for α- and β-Thalassaemia Alleles

α-thalassaemia genotypes were tested using 13 different determinants commonly found in Malaysia and those reported from the Southeast Asia region. The panel included four double α globin gene deletions (−−^SEA^, −−^MED^, −−^FIL^, and −−^THAI^), three single gene deletions (−α^3.7^ rightward deletion, −α^4.2^ leftward deletion, and −α^20.5^) and six non-deletion α2 globin gene mutations namely initiation codon (ATG > A-G), codon 30 (ΔGAG), codon 35 (TCC > CCC), codon 59 (GGC > GAC), codon 125/Hb Quang Zhe (CTG > CCG) and termination codon/Hb Constant Spring (TAA > CAA). In-house optimised multiplexed Gap-PCR and multiplexed amplification refractory mutation system (M-ARMS) methods were used in parallel for gene deletion and mutation testing, respectively [11,19,20].

For β-thalassaemia genotyping, 20 different mutations were tested: 19 by M-ARMS and one by simple ARMS technique. Genotyping of the 19 β-thalassaemia determinants were carried out in five separate multiplexed PCR reactions based on the shared thermo-cycling conditions of respective allele-specific primers as described by Hassan *et al.* (2013) with slight modification [13]. In the first M-ARMS-A reaction, allele specific primers for four mutations IVS 1-5 (G > C), Cd 41/42 (−TTCT), Cd 17 (A > T) and Cd 26 (G > A) were multiplexed, while IVS 1-1 (G > T), Cd 8/9 (+G), −28 (A > G) and Cd 71/72 (+A) mutations were amplified in M-ARMS-B reaction. In the third M-ARMS-C reaction, alleles IVS 1-1 (G > A), Cd 43 (G > T), Cd 16 (−C), and Poly A (A > G) were multiplexed, while M-ARMS-D had allele-specific primers targeted at −88 (C > T), initiation codon (ATG > AGG), Cd 15 (G > A) and −29 (A > G) mutations. Finally, allele specific primers used in M-ARMS-E reaction were to screen mutations −86 (C > G), Cd 19 (A > G) and Cap + 1 (A > C) [13]. IVS 2-654 (C > T) was tested in a separate ARMS reaction.

### 4.4. Gel Electrophoresis

The PCR products for α-thalassaemia were analysed by using agarose gel electrophoresis. PCR products of multiplex gap PCR were run on a 1.5% agarose gel electrophoresis using 1× Tris-Borate-EDTA (TBE) buffer. Whereas, PCR products for α-thalassaemia from multiplex ARMS PCR were run on a 1.2% agarose gel.

The PCR products from the β globin gene, except for reaction in MARMS-A, MARMS-B and MARMS-F, were separated using 2% agarose D1, Low EEO Pronadisa (Laboratorios CONDA, Madrid, Spain) in 1X TBE buffer (Biobasic, Markham, ON, Canada). DNA band was visualized under transilluminator (Vilber Lourmat, Sud Marne-la-Vallée, France). QIAxel Advanced System (QIAGEN GmBH, Hilden, Germany) was used to analyse MARMS-A, MARMS-B, and MARMS-F which used automated capillary electrophoresis to separate the products based on their sizes and visualised on the interfaced computer system using its dedicated QIAxcel ScreenGel Software (QIAGEN GmBH, Hilden, Germany).

## 5. Conclusions

In this study, we have demonstrated the α- and β-thalassaemia gene frequency in the Penang population and further described its allelic distributions in the Malay populations. The heterogeneity of the α globin defects in the present study population is different from the other states in Malaysia as well as the presence of rare types of β-thalassaemia mutations. This may be attributed to a unique situation where there is a higher proportion of miscegenation among Malay with other ethnicities.

The results from this study will serve as a baseline for further investigations into the genetic defects. This information would provide health care professionals better awareness of the possible clinical spectrum of α- and β-thalassaemia in this geographic locality, thus facilitating better diagnostic and management of the disease. The prevention of severe α- and β-thalassaemia syndrome is very much dependent upon the availability of molecular characterisation, supported by adequate genetic counselling, and targeted public awareness programmes. The molecular diagnosis of α- and β-thalassaemia patients followed by genetic counselling of at risk couples should be made available as it is essential for the accurate diagnosis of both carrier and disease states.

In addition, such an effort would reduce the economic burden, and comprehensive and effective management of this problem in our country will be better achieved. Thus, a coordinated and interactive collaboration between the relevant stakeholders is necessary to ensure the effectiveness and success of the National Prevention and Control Programme for Thalassaemia in Malaysia.

## Figures and Tables

**Table 1.** Spectrum of α-thalassaemia gene defects identified in Malay patients by multiplex Gap- and ARMS-PCR.

Alleles	No. of Mutations	Prevalence (%)
−−^SEA^	20	35.7
α^Constant Spring^α	17	30.3
−α^3.7^	10	17.9
Cd 59 (GGC > GAC)	6	10.7
αα haplotype	3	5.4
Total	56	100

**Table 2.** Prevalence of α-thalassaemia genotypes in Malay patients.

Genotypes	No. of Patients *n* = 28	Prevalence (%)
−−^SEA^/α^CS^α	13	46.4
−α^3.7^/−−^SEA^	6	21.4
α^Cd 59^α/α^CS^α	4	14.3
−α^3.7^/−α^3.7^	2	7.1
α^Cd 59^α/αα	2	7.1
−−^SEA^/αα	1	3.6
Total	28	100

**Table 3.** Frequency of β-thalassaemia mutations identified in Malay patients using Multiplex-ARMS.

Mutations	Percentage % (*n*)	Total
Heterozygous mutation		
β/β^Cd 26 (G-A)^	55.6 (5)	
β/β^IVS 1-1 (G-T)^	22.2 (2)	
β/β^Cd 41/42 (−TTCT)^	22.2 (2)	9
Compound heterozygous		
β^Cd 26 (G-A)^/β ^IVS 1-5 (G-C)^	32.1 (9)	
β^Cd 26 (G-A)^/β^IVS 1-1 (G-T)^	17.8 (5)	
β^Cd 26 (G-A)^/β^Cd 41/42 (−TTCT)^	14.3 (4)	
β^Cd 26 (G-A)^/β^Cd 17 (A-T)^	7.1 (2)	
β^Cd 26 (G-A)^/β^Cd 15 (G-A)^	7.1 (2)	
β^Cd 26 (G-A)^/β^Cd 8/9 (+G)^	3.6 (1)	
β^Cd 41/42 (−TTCT)^/β^Cd 19 (A-G)^	3.6 (1)	
β^Cd 41/42 (−TTCT)^/β^-28 (A-G)^	3.6 (1)	
β^Cd 41/42 (−TTCT)^/β^IVS 1-1 (G-T)^	3.6 (1)	
β^IVS 1-5 (G-C)^/β^IVS 1-1 (G-T)^	3.6 (1)	
β^IVS 1-5 (G-C)^/β^Cd 19 (A-G)^	3.6 (1)	28
Uncharacterised		3
Total		40

**Table 4.** Distribution of β-thalassaemia mutations identified in Malay patients.

Type of Mutations	Number (*n*)	Prevalence (%)
Cd 26 (G-A)	28	43.1
IVS 1-5 (G-C)	11	17.0
IVS 1-1 (G-T)	9	13.8
Cd 41/42 (−TTCT)	9	13.8
Cd 15 (G-A)	2	3.1
Cd 17 (A-T)	2	3.1
Cd 19 (A-G)	2	3.1
Cd 8/9 (+G)	1	1.5
−28 (A-G)	1	1.5
Uncharacterised	6	
Wild type	9	
Total	80	100
